# Dog ownership for people with substance use disorder: self-reported influence on substance use and mental health

**DOI:** 10.1186/s13011-025-00653-x

**Published:** 2025-06-19

**Authors:** Andi Kerr-Little, Jørgen G. Bramness, Ruth C. Newberry, Stian Biong

**Affiliations:** 1 Norwegian National Advisory Unit on Concurrent Substance Abuse & Mental Health, Disorders, Oslo, Hamar Norway; 2 Institute of Clinical Medicine, The Arctic University of Norway (UIT), Tromsø, Oslo, Norway; 3 Department of Alcohol Drug and Tobacco Research, Norwegian Institute of Public Health, Oslo, Norway; 4 Section for Clinical Addiction Research, Oslo University Hospital, Oslo, Norway; 5 Department of Animal & Aquacultural Sciences, Faculty of Biosciences, Norwegian University of Life Sciences, ÅS, Oslo, Norway; 6 Lovisenberg Diaconal University College, Lovisenberggata 15B, Oslo, 0456 Norway

**Keywords:** Substance use, Dog ownership, Human-animal interaction, Mental health, Qualitative methods

## Abstract

**Background:**

Dog ownership has been reported to positively influence the lives of individuals with substance use disorder (SUD) fostering social connection, a sense of belonging, and greater daily structure. However, the specific ways in which dog ownership impacts substance use and mental health remain underexplored. This study aimed to explore how people with SUD perceived that dog ownership affected their use of substances and mental health characteristics.

**Method:**

Qualitative, semi-structured, in-depth interviews were conducted with eight individuals with experience of dog ownership and SUD. Data were gathered and analysed using a 4-step qualitative content analysis.

**Results:**

Three key categories emerged from the analysis. The unique relationship with their dog encouraged the development of a new sense of self for participants that had not been previously possible. Participants reported an increased awareness and regulation of substance use, and they became more mindful of their use, often reducing or managing it to align with caring for their dog. The bond with their dog contributed to improved mental health, emotional stability and appeared to play a role in reducing suicidal ideation.

**Discussion:**

Dog ownership provided participants with a positive sense of self and reinforced feelings of self-worth. This helped them move away from impulsive or habitual substance use patterns and adopt a more intentional, less harmful approach. The relationship with the dog also appeared to stabilise participants’ mental health, enabling them to navigate depressive episodes more effectively and recover from negative moods more easily.

## Introduction

Substance use disorder (SUD) is a complex condition frequently associated with serious physical health conditions such as heart disease, cancer, liver disease, and sexually transmitted infections (Schulte and Hser 2013). SUD commonly co-occurs with psychiatric disorders including depression, anxiety disorders, bipolar disorder, antisocial personality disorder and post-traumatic stress disorder (PTSD) (Conway et al. 2006; Magidson et al. 2012; Reynolds et al. 2005; Torrens et al. 2011). This relationship complicates treatment, necessitating integrated approaches that address both substance use and mental health simultaneously (Drake et al. 2004; Hasin et al. 2002; Ruppelt et al. 2020; Samet et al. 2013). Given the chronic nature of SUD, treatment models that emphasize ongoing support and the empowerment of individuals in managing their condition have been associated with improved outcomes, including higher rates of sustained abstinence and moderate use, and enhanced long-term recovery stability (Beaulieu et al. 2021; White & Kelly 2011). This approach emphasizes strategies that promote long-term stability and personal empowerment while living with a chronic condition (Lenaerts et al. 2014).

Whereas traditional recovery frameworks prioritise *human* relationships and clinical interventions, there is increasing recognition of the role that non-human connections can play in supporting recovery. Animal-assisted services (AAS) involving dogs have been associated with benefits to individuals with SUD by alleviating cravings for substances (Contalbrigo et al. 2017; Husband et al. 2020), improving the therapeutic alliance between patient and clinician (Wesley et al. 2009), and increasing engagement in treatment (Trujillo et al. 2020). These findings suggest that building a caring relationship with a dog could positively influence how substance use treatment is perceived and engaged with by individuals with SUD.

Beyond formal treatment settings, dog ownership can play a role in supporting individuals through their substance use journey (Dell et al. 2023; Kerr-Little et al. 2023; Kosteniuk and Dell 2020). Dogs provide a unique form of non-judgmental emotional support, helping individuals with SUD who may experience social isolation and stigma (Irvine 2013; Kerman et al. 2019; Kerr-Little et al. 2023). For participants in a methadone maintenance programme, dog ownership was reported to positively impact social and home environments, enhance health and well-being, and instil a sense of purpose (Kosteniuk and Dell 2020). Despite these benefits, mechanisms by which dog ownership impacts substance use behaviour itself remain underexplored.

For individuals with serious mental health conditions, pet ownership has been shown to be associated with feelings of love and connection, which were linked to reduced symptom severity and improved self-worth (Hawkins et al. 2021; Hui Gan et al. 2020; Hunt and Stein 2007; Wisdom et al. 2009). Pets are often viewed as part of the core support system (Brooks et al. 2019) and their presence may serve as a valuable resource for enhancing emotional well-being and supporting mental health recovery.

Limited evidence also suggests that dog ownership may motivate behavioural change. Individuals experiencing homelessness, for example, have cited dog ownership as a driver in decisions to leave the streets and adopt less harmful behaviours (Bender et al. 2007; Thompson et al. 2006). Additionally, people experiencing homelessness reported less drug use after becoming dog owners, with this change linked to the desire to care for their dog or fear of losing the dog (Kerman et al. 2019). However, owning a dog may also exacerbate vulnerable states, such as increasing substance use in response to the emotional distress associated with pet loss (Thompson et al. 2016) or by limiting housing options due to restrictive pet policies, which present a barrier specifically for those with companion animals (Slatter et al. 2012a).

From a harm reduction perspective, relationships between dog ownership, mental health and substance use behaviour merit further examination. Harm reduction strategies focus on incremental improvements in substance-related behaviours and overall well-being, particularly for individuals managing long-term addiction (Hawkins et al. 2017; MacMaster 2004; Tatarsky and Marlatt 2010). In this context, the responsibilities of dog ownership may support behavioural changes by encouraging greater stability, social connectedness and a sense of belonging. Owning a dog may gradually reduce harmful behaviours as individuals stabilise their everyday lives to care for their dog (Kerr-Little et al. 2023).

Existing research has explored the benefits of animal-assisted services (Contalbrigo et al. 2017; White & Kelly 2011) and the role of pet ownership among people experiencing homelessness (Slatter et al. 2012b). Nevertheless, scarce research has directly examined the relationship between dog ownership and its impact on substance use regulation and mental health. To address this gap, a qualitative study was conducted where we aimed to explore and deepen our understanding of how dog owners with SUD perceive that their dog impacts their mental health and use of substances. We aimed to examine specific ways in which dog ownership was reported to affect substance use and influence mental health. Our research question was `How do dog owners living with SUD experience and describe the influence of their dog on their substance use and mental health?´.

## Materials and methods

### Design

To gain insight into human experiences, we adopted a qualitative approach suitable for under-researched areas where limited theory exists (Sandelowski 2000). Drawing on a phenomenological perspective, we used a descriptive exploratory design to foreground participants’ lived experiences while minimising researcher interpretation. Given the limited knowledge in this area, our aim was to stay as close as possible to participants’ directly reported accounts. To achieve this, we employed semi-structured interviews with people who had experience of dog ownership and SUD. Content from these interviews was previously evaluated in a study focussing on the relationship between dog ownership and the everyday lives of people with SUD (Kerr-Little et al. 2023), indicating that owning a dog resulted in feeling more socially connected and added more structure to the day. In the current analysis, we focused directly on how they reported changes in their mental health and substance use patterns.

The study was conducted in collaboration with representatives of services working with people with SUD. A user representative (Beresford 2013) with personal experience of both SUD and dog ownership was included in the research team. This collaborative approach ensured that questions were grounded in practice-based relevance and that the study reflected the authentic voices and experiences of individuals living with SUD. The questions focused on broad areas of dog ownership such as how the participants had been affected by owning their dog and how the dog impacted their substance use. The rationale guiding the development of questions was to allow participants to reflect freely on how dog ownership related to their mental health and substance use.

Meetings with those working within the SUD field were conducted as part of the preparations for the study, with the user representative being consulted regarding the planning, development, recruitment and discussion of results.

### Participants

We recruited participants in Oslo, Norway by distributing information pamphlets at low threshold centres (Edland-Gryt and Skatvedt 2013). In addition, the first author (AKL) participated in an outreach programme to facilitate inclusion of participants who were not in treatment. The sampling strategy was purposeful, aiming for inclusion of diverse participants in terms of current substance use, age and gender (Seidman 2013). The sample size was determined by weighing the burden of participation by members of a sensitive population against the quality of information gathered (Guest et al. 2006; Hagaman and Wutich 2017). After 8 in-depth interviews, we found we had rich data for analysis. Participants were required to have experience of dog ownership while actively engaging in substance use. To enable gathering of a broad range of experiences, current dog ownership and currently active substance use were not requirements.

Eight participants took part in the project (5 females and 3 males), with ages ranging from 36 to 54 years. All participants stated that they had had periods of substance use that had negatively affected their lives. The main drugs reported were heroin (8 people), amphetamine (2 people) and cannabis (2 people). One participant reported no current substance use, while the other 7 reported varying severities of substance use. Four participants were in opiate (methadone) maintenance treatment. All participants lived alone, and all claimed benefits as their main source of income. Six lived in rental property, one lived in an institution, and one was homeless at the time of interview. Each participant referred to a single dog that they had owned for an average of 5.9 years (range: 1.5–14 years). All dogs were pet dogs of unstated origin with no special training for task-related services or otherwise. They included 3 mixed breed dogs, 2 rottweilers, 2 collies and a poodle. Two participants were not current dog owners but had owned a dog within the last year.

Participants were given a written summary of the project, which was read to them at the place of interview. This included informing them that they could withdraw from the project at any time and that their identity would remain anonymous. Informed consent was obtained from each participant before starting the interview. All information obtained was self-reported by the participants, and the authors did not have access to any records about the participants.

### Data collection

We collected data using semi-structured, in-depth individual interviews (Brinkmann and Kvale 2015). The questions gave direction but were open-ended to allow the participants space to reflect on their lived experiences and expand as required (Seidman 2013). The interviews took place at an open use centre, except for one at an opiate maintenance centre and one in the participant´s home for mental health reasons. Five participants were accompanied by their dog during the interview. The interviews were conducted and audio-recorded by AKL in a quiet location, with pauses made if required, and took from 45 to 90 min to complete. A gift voucher was provided in return for participating in an interview. Audio recordings were stored on a secure server and transcribed by AKL.

### Analysis

We applied a 4-step qualitative content analysis centred on the manifest content in the interview text (Graneheim and Lundman 2004). An overall impression of the text was achieved by reading through the transcripts several times. This reading allowed identification of meaning units relevant to substance use and mental health, which were sentences or a collection of sentences containing content relevant to addressing the research question. In Step 2, these meaning units were condensed into shorter form. Similar reported experiences across participants were organised into subcategories (Step 3), which were then integrated into broader categories (Step 4). The content analysis was performed by AKL, with consensus on the categorisation arrived at by conferring with the co-authors and user representative (see Table 1 for an example of this categorisation process).

**Table 1:** Example showing how interview text was condensed into the category: “the dog influenced me as no one else could.”

Meaning Unit	Condensed meaning unit	Subcategory	Category
He meant everything and has influenced me in a way that no one else could’ve influenced me. I think if I had had a girlfriend, it would’ve probably been someone who took drugs so it wouldn’t have influenced me in the same way.	My dog influenced me as no one else could.	My dog influenced me differently to how people influence me.	My dog influenced me as no one else could.
He made me into a better person…in every way, to not do drugs, not be angry, to take responsibility, to give love, to be social.	He made me into a better person.	I felt I changed as a person because of my dog.	
She is me. I see that we are very similar. I see that we are very stubborn. I see myself when I see her.	I see myself when I see her.	There´s someone else like me.	

## Results

Table 2 outlines categories and sub-categories formulated from participant responses, gaining a deeper understanding of how marginalized individuals with SUD perceived and described the impact of dog ownership on their mental well-being and substance use patterns.

**Table 2:** Categories and subcategories

Sub-Category	Category
My dog influenced me differently to how people influence me.	My dog influenced me as no one else could.
I felt I changed as a person because of my dog.	
There´s someone else like me.	
I don’t take drugs the way I did before.	Because of my dog, I don’t take drugs the way I did before.
I didn’t want my dog to see me using drugs.	
I prioritised my dog.	
I must be mentally stable for my dog.	My dog meant a lot to me psychologically.
She meant a lot to me psychologically.	
I realised I was important.	
At times I worry.	

Owning a dog appeared to be influential in affecting how the participants related to their substance use and mental health. Three categories of responses emerged from the qualitative analysis of interview content: `My dog influenced me as no one else could´ `Because of my dog, I don´t take drugs the way I did before´ and `My dog meant a lot to me psychologically´.

### My dog influenced me as no one else could

This category addresses how participants felt that the influence of the dog was distinct and unique precisely because it had come from a dog. The bond with their dog created something different in their lives compared with their previous experience.

For some, the influence of the dog went beyond what they felt a partner or family member could have offered. One participant shared that if the relationship had come from a partner, then they would most likely have taken drugs together, whereas this was not the case with the dog.

*`He meant everything and has influenced me in a way that no one else could’ve influenced me. I think if I had had a girlfriend*,* it would’ve probably been someone who took drugs so it wouldn’t have influenced me in the same way.´ Male*,* 54 years old*.

Others highlighted how caring for a dog was different from the experience of raising a child. One participant described how overwhelming it had been to care for her daughter. Yet, with her dog, she developed a relationship that allowed her to go at her own pace, offering responsibility without the pressure she had felt as a parent.

*`The most important is that I could take drugs. I couldn’t take care of my daughter but with a dog*,* you know*,* there is a lot that has fallen into place. I can take things at my speed. I need that. I couldn’t do that with my daughter. It’s not that I can gamble with [dog’s name]. That’s not what I mean*,* but the demand is not the worst*,* and the pressure is not so big but at the same time you have the responsibility and I need that.´ Female*,* 48 years old*.

Participants spoke of getting their dog as a turning point, describing changes in themselves that they had not anticipated. Getting a dog gave participants a new way of relating to themselves, helping them feel more in control of themselves and how they related to their lives. They described how this affected even personal qualities within themselves, from describing changes in lifestyle to becoming more trustworthy and thinking more constructively.

*`He made me into a better person…in every way*,* to not do drugs*,* not be angry*,* to take responsibility*,* to give love*,* to be social.´ Male*,* 36 years old*.

For some participants, the dog served as a mirror of themselves, stating that they could see themselves in their dog. The dog often reflected the participants’ own emotions back to them, validating their experience and reinforcing a sense of self-acceptance.

*`She is me. I see that we are very similar. I see that we are very stubborn. I see myself when I see her. Female*,* 40 years old*.

The relationship with the dog was described as distinct from any human relationship. It provided a new way of seeing themselves, offering participants an emotional connection that was entirely separate from their substance use. For many, the connection with the dog was not just about companionship; the relationship opened a space that was different to their experience of human relationships and so was able to impact their substance use and mental health in ways that had not been possible previously.

### Because of my dog, I don´t take drugs the way I did before

This category represents how participants described changes in their substance use patterns after becoming dog owners. They directly attributed a reduction in their drug use to their dogs. Participants discussed how the bond with their dog fostered a self-awareness about their drug use, making them more mindful of how much and what kind of substances they used.

Frequently participants explained how their drug use became more managed. Many participants described having better control of their drug taking and not wanting to take drugs in the presence of their dog. For example, one participant described how before getting the dog, she would go to the park and use drugs, whereas after getting the dog she was able to go to the park without using substances as she did not feel it was right to do so when she had her dog with her. Another participant emphasised the importance of having a dog sitter if he wanted to use drugs, as he did not want his dog to see him taking drugs.

Participants explained how they changed which drugs they used, moving to substances which they believed gave a more predictable outcome, so that they did not risk becoming unable to care for the dog. One participant described moving to less destructive means of drug taking, including moving from injection use to smoking.

*`I don’t take drugs the way I did before I got her… I must be awake and present. There is less drugs… It’s not that I don’t do any drugs. No. I’m just more careful*,* more respectful. I care that she does not have a bad experience… My drug use has become much better. I cut out injecting.´ Female*,* 40 years old*.

*`I don’t use amphetamine or pills anymore as I don’t know which direction it’s going to go in. I’d be concerned that I couldn’t take care of him*,* and that care giving’s really good for my mental health.´ Female*,* 48 years old*.

One of the participants described how becoming a dog owner led him to stop his drug use entirely. He had been afraid that if he continued, then he would end up losing the dog, so he decided to stop his use altogether.

Participants stated not wanting to be seen by their dog whilst using drugs as the reason why the dog had impacted their drug use. Being seen by the dog triggered a sense of reflection for the participants and appeared to prompt self-conscious feelings regarding their substance use.

*`I don’t want her to see me completely wasted´ female*, *42 years old*.

In summary, after becoming dog owners, participants reported a reduction in drug use, and a greater awareness of the substances they used. In some cases, they reported a shift away from unpredictable or harmful substances or methods of use. Not wanting to be seen by, or not being able to care for, their dog appears to have been a primary driver in fostering more intentional and safer substance use behaviours.

### My dog meant a lot to me psychologically

This category highlights how participants’ relationship with their dog influenced their mental health. Forming the relationship with their dog fostered a sense of emotional understanding and self-worth. Participants often shared how their dog helped them feel valued and important, even during times of psychological distress.

The dogs’ unconditional acceptance impacted participants’ mental health. The absence of judgment or stigma in their dog’s presence provided a safe emotional space, allowing participants to feel valued and appreciated as they were. Many described this relationship using words like “respect” and “gratitude,” underscoring how they believed their dog viewed them in a positive and affirming way. Participants felt emotionally understood by their dogs, which helped them feel that their experiences and emotions had a place in life and were acknowledged. This bond nurtured a sense of psychological safety and self-worth.

*`It affected me mentally too. She was my best friend. She meant a lot to me psychologically.´ Male*,* 50 years old*.

*`Really a support because*,* as I said*,* she got me to go out but not just that. She knew if I was sad.´ Female*,* 53 years old*.

At times, it appeared the simple interactions with the dog changed the state of mind for participants. The playful, joyful presence of the dog created positive change.

*`He is everything*,* always so happy. Even if you are in a bad mood*,* he’ll come over and try and comfort you to get you in a better mood. He tries everything to be fun. Come on*,* be in a better mood. That’s it. I’ll be really captivated*,* he’ll win the argument*,* and that’s good.´ Male*,* 54 years old*.

Participants spoke of how they experienced being stuck in periods of depression before owning their dog, and how this shifted after getting a dog. They spoke of the love and companionship the dog provided which enabled them to get through “mentally tough and evil days”. Acknowledging that experiencing challenging mental periods previously led to increased substance use, they felt that being with the dog helped prevent their mental state becoming so low and thus stabilised their subsequent substance use.

*`Before I had a dog I was depressed*,* I didn’t want to go out. I wasn’t interested in living´ Male*,* 36 years old*.

*`My dog meant everything for me. The only thing I was doing was taking care of her. Had I not had my dog*,* I could’ve ended up in the river down there. I was depressed and completely down´ Male*,* 50 years old*.

The bond they formed with their dog was described as lifesaving, with some participants reflecting that their dog played a crucial role in preventing suicidal thoughts. One participant credited his dog with keeping him alive, while another described his dog as “everything” during a period of depression that previously would have led to suicidal tendencies. The bond and the sense of being needed by their dog appeared important when considering how the dog had influenced them mentally.

*`I have had some suicide attempts the last few years but never while I had the dog. How could I? I couldn’t take my own life. I couldn’t hang myself in the kitchen*,* to put it like that*,* and have the dog locked in. There was no-one that knocked on the door every day*,* so the dog would suffer over it*,* so it wasn’t appropriate*,* and the dog came first. If I had not had her*,* I probably would have considered taking my own life more times than I had done already. Yeah*,* it wasn’t even a topic when I had her. I didn’t do it because then she would be standing alone. She wouldn’t manage alone.´ Male*,* 50 years old*.

Whilst overwhelmingly positive, dog ownership also presented challenges to participants´ mental health. One participant described how she felt more paranoid due to the worry that someone might steal her dog, whilst another participant described how after her dog got lost, she increased her substance use, before reducing it again to enable her to look for him.

In conclusion, participants’ relationship with their dog provided critical support for their mental health, helping them navigate feelings of self-worth, move out of depressive episodes and combat the challenges of suicidal tendencies. Through this bond, participants were able to develop a more positive self-view and gain emotional stability. However, dog ownership was not without challenges, as the responsibility and attachment to their dog occasionally brought additional worries.

## Discussion

This study investigated how people with SUD perceived their dog to influence their substance use and mental health. The findings suggest that participants found the bond with their dog unique and, as such, able to influence them differently from the way a human relationship could, allowing for personal growth. They described how dog ownership led to a reduction in substance use and played a key role in fostering a sense of value and worth. Participants described a stabilising of their mental health, with benefits ranging from shifting out of a negative mood to preventing depressive episodes and suicidal ideation.

### Unique relationship

Participants consistently emphasised the unique nature of their relationship with their dog, often describing the bond as central to how they saw themselves. This transformation in self-concept can be understood through symbolic interactionist theory, which holds that identity is shaped through relational processes and the internalisation of others’ perceptions (Mead and Morris 1934). Within this framework, companion animals are recognised as “significant others” who participate in these interactions. Sanders (1990) and Irvine (2004) argue that animals can function as extensions of the self, helping individuals construct and reinforce a socially defined identity. Brown (2007) similarly describes pets as self-objects that support the development of a cohesive sense of self. These frameworks help explain why the relationship with a dog may be especially meaningful for individuals with SUD, as it offers a stable and affirming relational context that contributes to self-worth and identity formation.

The relevance of these theoretical frameworks was evident in participants’ narratives, which illustrated how the bond with their dog shaped their self-perception and facilitated processes of personal growth. The bond with their dog provided a unique avenue for participants to foster a renewed sense of self. However, it remains unclear whether individuals who are already more stable and capable of caring for a dog are more likely to acquire one, or if it is the relationship with the dog itself that drives this stabilisation. Humans naturally seek to attribute meaning and causality to their experience to make sense of their lives. The strong bond with their dog may have prompted participants to construct narratives in which their dog played a central role in driving positive change, aligning with the tendency to create coherent narratives that connect life events into a meaningful framework (Bruner 1987). Nevertheless, the construction of a coherent sense of self through narrative has been shown to enhance self-understanding (McAdams 1996), and is essential in building psychological wellbeing and resilience (Griffiths 2009; Waters and Fivush 2015).

### Substance use

SUD is often associated with risky behaviours and impulsive substance use, but the degree of risk varies significantly depending on individual circumstances (Pates and Riley 2012). From a harm reduction perspective, even small reductions in substance use or shifts towards less harmful approaches are seen as positive (MacMaster 2004; Tatarsky 2003; Tatarsky and Marlatt 2010). In this study, participants consistently reported that owning a dog influenced them to reduce or modify their substance use, stating that they opted for safer alternatives in terms of how they used substances or what they used. Participants describe moving away from injection use and, by such, lowering their risk of overdose or infection (Bridge 2010; Carico et al. 2020; Darke 2003; Novak and Kral 2011; Pates and Riley 2012). They also reported a shift toward more intentional and planned substance use, a departure from the impulsive patterns often seen in SUD use (de Wit 2009; Moeller and Dougherty 2002; Tarter et al. 2007; Verdejo-García et al. 2008). This shift may be interpreted through the lens of the relational bond participants formed with their dog, marked by a desire to remain present, to avoid being perceived as intoxicated or erratic in their dog’s presence, or to uphold a sense of responsibility in the daily care. These relational motivations appeared to foster more intentional decision-making regarding substance use, and grounded a commitment to maintain a stable and attentive role in the dog’s life.

Becoming a dog owner appeared to foster self-awareness in participants, as they saw themselves as influential and valued in their dog’s life. This relationship acted as a catalyst for more deliberate decision-making, reducing impulsive substance use and promoting safer behaviours.

### Mental health

Participants highlighted the impact their dog had on their mental health. The dog´s acceptance provided a safe, judgment-free emotional space, which helped develop a sense of self-worth and psychological stability. Solberg and Nåden (2020) emphasise that being met with an open and non-prejudiced attitude is essential for dignity and human wholeness and worth (Edlund et al. 2013). Through their relationship with their dog, participants reported feeling accepted and loved, which contributed to a stronger sense of self-worth.

Interacting with dogs has been shown to increase positive affect and aid in emotional regulation (Madden Ellsworth et al. 2016; Matijczak et al. 2024; VanFleet and Faa-Thompson 2010). Participants in this study echoed these findings, describing how the joy and happiness their dog brought into their lives helped them to shift from a negative to a positive mood. Playful interactions with their dog served as a means to reduce stress and redirect their focus away from substance use.

Participants also described experiencing fewer and less intense depressive episodes after owning a dog. This aligns with research showing that interaction with a dog can reduce symptoms of depression and negative mood (Crossman et al. 2015; Stern et al. 2013). However, the relationship between dog ownership and mental health is complex, as large-scale demographic studies sometimes report mixed findings, suggesting that further research is needed to clarify specific mechanisms (Merkouri et al. 2022).

Although participants were not explicitly asked about suicidal thoughts, many voluntarily shared such experiences when discussing their dog. Several participants credited their dog with saving their life, describing how the responsibility and companionship of dog ownership prevented them from acting on suicidal ideation. Mental illness, including SUD and major depression, is a well-established risk factor for suicide.

(Darke and Ross 2002; Favril et al. 2022; Fu et al. 2023; Wilcox et al. 2004), with psychological factors like hopelessness (Zhang and Li 2013) and mental pain (Verrocchio et al. 2016) also playing a significant role. Participants appeared to describe their dog as an emotional anchor during moments of distress. This finding supports previous research suggesting that dogs can act as a protective factor against suicide by fostering connection and interrupting distressing thoughts (Stern et al. 2013).

However, the dependency on the dog also appeared to introduce vulnerabilities, including heightened anxiety about the dog’s well-being and increased substance use during particularly challenging times. These findings highlight both the protective benefits and potential emotional risks of dog ownership, suggesting that, while dogs may provide critical support, they can also contribute to emotional strain in specific situations.

### Limitations and future directions

Although our sample size was limited, the participants´ rich descriptions and nuanced narratives provided in-depth insight into the complex interplay between dog ownership, substance use, and mental health within the context of the Norwegian welfare state. Specifically, the study illuminated how the perceived responsibility of caring for a dog contributed to reductions in high-risk behaviours, fostered a sense of self-worth, and promoted mental wellbeing and stability, factors which are often protective in substance use recovery. These insights are particularly relevant for service providers aiming to develop trauma-informed, person-centred interventions that take into account the stabilising role of meaningful relationships, including those with companion animals. The findings also highlight the need to consider the relational context of recovery in future policy and programme development. This exploratory work lays the groundwork for further research examining longitudinal changes, the impact of dog ownership or dog loss at various recovery stages, and the experiences of individuals for whom dog ownership is less beneficial. Additional research could also investigate how variables such as participant age or gender, and the type of companion animal, influence outcomes to determine whether different animals produce similar or distinct effects and to inform more tailored interventions.

### Animal welfare considerations

While this study has focused on the impact of dog ownership on participants’ mental health and substance use, an important but often overlooked aspect is the welfare of the dog itself. Participants’ accounts revealed both challenges and efforts in ensuring their dog’s well-being, highlighting the complexities of caregiving within the context of substance use. Despite these concerns, direct observations from AKL during the interviews suggested that the dogs were generally well cared for, sociable, and not fearful. The strong emotional bond between participants and their dogs was evident, with owners displaying genuine affection and attentiveness. The dogs appeared happy, friendly, and comfortable in their interactions with their owners, AKL and others.

## Conclusions

This study highlights the potential for dog ownership to positively influence substance use behaviour and mental health characteristics among individuals with SUD. By fostering a sense of self-worth, reducing impulsive behaviour, and providing emotional stability, dogs can serve as powerful allies in recovery. Understanding the importance of relationships with pet dogs and the influence they can have on both mental health and substance use patterns opens the possibility for a more tailored approach to the treatment of SUD in dog owners. The study also illuminates how the connection with a dog can encourage development of a sense of self and a move toward a less harmful way of living.

## Data Availability

The dataset analysed in the current study is available from the corresponding author upon reasonable request.

## Abbreviations

SUD: Substance use disorder
